# The Aryl Hydrocarbon Receptor-Activating Effect of Uremic Toxins from Tryptophan Metabolism: A New Concept to Understand Cardiovascular Complications of Chronic Kidney Disease

**DOI:** 10.3390/toxins6030934

**Published:** 2014-03-04

**Authors:** Marion Sallée, Laetitia Dou, Claire Cerini, Stéphane Poitevin, Philippe Brunet, Stéphane Burtey

**Affiliations:** 1 Aix Marseille Université, Inserm, VRCM, UMR_S 1076, Marseille13005, France; E-Mails: marionsallee@yahoo.fr (M.S.); claire.cerini@univ-amu.fr (C.C.); stephane.poitevin@univ-amu.fr (S.P.); drphilippe.brunet@outlook.com (P.B.); stephane.burtey@ap-hm.fr (S.B.); 2 Assistance Publique Hôpitaux de Marseille, CHU Conception, Centre de Néphrologie Dialyse Transplantation Rénale, Marseille 13005, France

**Keywords:** tryptophan-derived uremic toxins, aryl hydrocarbon receptor, chronic kidney disease, cardiovascular diseases

## Abstract

Patients with chronic kidney disease (CKD) have a higher risk of cardiovascular diseases and suffer from accelerated atherosclerosis. CKD patients are permanently exposed to uremic toxins, making them good candidates as pathogenic agents. We focus here on uremic toxins from tryptophan metabolism because of their potential involvement in cardiovascular toxicity: indolic uremic toxins (indoxyl sulfate, indole-3 acetic acid, and indoxyl-β-d-glucuronide) and uremic toxins from the kynurenine pathway (kynurenine, kynurenic acid, anthranilic acid, 3-hydroxykynurenine, 3-hydroxyanthranilic acid, and quinolinic acid). Uremic toxins derived from tryptophan are endogenous ligands of the transcription factor aryl hydrocarbon receptor (AhR). AhR, also known as the dioxin receptor, interacts with various regulatory and signaling proteins, including protein kinases and phosphatases, and Nuclear Factor-Kappa-B. AhR activation by 2,3,7,8-tetrachlorodibenzo-p-dioxin and some polychlorinated biphenyls is associated with an increase in cardiovascular disease in humans and in mice. In addition, this AhR activation mediates cardiotoxicity, vascular inflammation, and a procoagulant and prooxidant phenotype of vascular cells. Uremic toxins derived from tryptophan have prooxidant, proinflammatory, procoagulant, and pro-apoptotic effects on cells involved in the cardiovascular system, and some of them are related with cardiovascular complications in CKD. We discuss here how the cardiovascular effects of these uremic toxins could be mediated by AhR activation, in a “dioxin-like” effect.

## 1. Introduction

Patients with chronic kidney disease (CKD) are at high risk for cardiovascular diseases. CKD leads to accelerated atherosclerosis and consequently to a marked increase in cardiovascular morbi-mortality [1,2]. For patients with CKD, the risk of dying prematurely of cardiovascular disease is much higher than the risk of progressing to dialysis or transplantation [3]. This higher risk cannot be totally explained by classical cardiovascular risk factors such as diabetes mellitus, tobacco use, hypercholesterolemia, high blood pressure, and obesity. The uremic environment itself is harmful and uremic toxins have emerged as a key factor to explain cardiovascular disease [4]. These uremic toxins are classified in three groups by their behavior during dialysis [5]: the small water-soluble molecules, the middle molecules, and the protein-bound molecules, which are hardly removed by conventional hemodialysis treatments. Among uremic toxins, those derived from tryptophan are of particular interest because they have cardiovascular toxicity and they are Aryl Hydrocarbon Receptor (AhR) ligands [6,7]. These toxins include toxins from the kynurenine and indolic pathways. This paper reviews how toxins derived from tryptophan can play a role in cardiovascular diseases associated with CKD via their property of AhR activation.

## 2. Uremic Toxins from Tryptophan Metabolism

**Figure 1:**
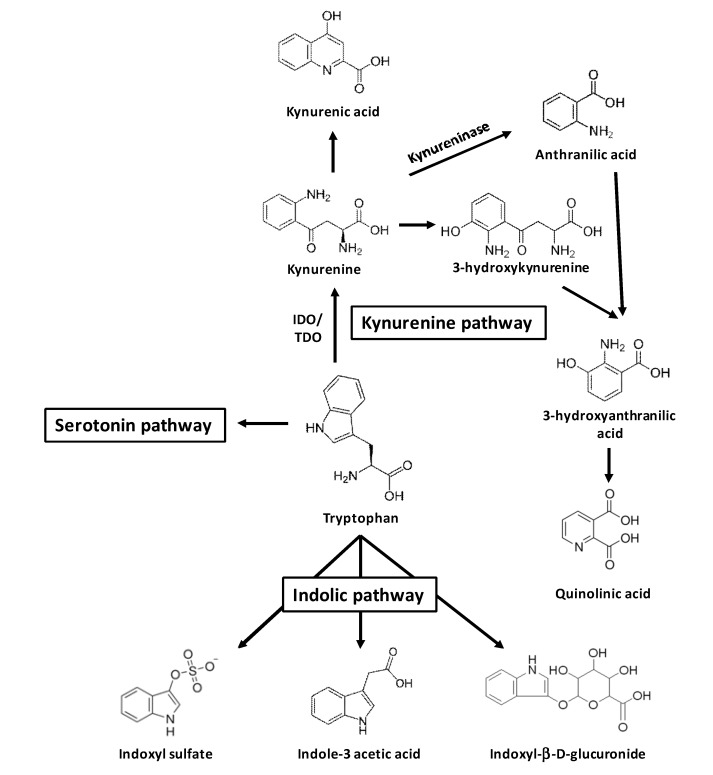
Uremic toxins from tryptophan metabolism.

Tryptophan is an essential amino acid found in the diet. Ninety-five percent of tryptophan can be metabolized through the kynurenine metabolic pathway (Figure 1). This pathway is mediated by the rate-limiting enzymes tryptophan 2,3-dioxygenase (TDO) and indoleamine 2,3-dioxygenase (IDO) [8]. TDO is highly expressed in the liver and is also found in the brain. Two isoenzymes of IDO exist, IDO-1 and IDO-2. IDO-1 expression is found in most tissues [8]. IDO activity leading from tryptophan to kynurenine is reflected by the tryptophan/kynurenine (TRP/KYN) ratio. In CKD patients, tryptophan serum level is decreased whereas metabolites of the kynurenine pathway, *i.e.*, kynurenine, 3-hydroxykynurenine, kynurenic acid, anthranilic acid, and quinolinic acid, are increased [9,10]. 

Two other metabolic pathways of tryptophan metabolism are also described: the serotonin pathway that leads to melatonin, and the indolic pathway that leads to indolic components such as indoxyl sulfate (IS), indole acetic acid (IAA), and indoxyl-β-D glucuronide (IDG) (Figure 1) [7,11,12]. Indolic components are produced by intestinal bacteria via tryptophan degradation before absorption [12]. Indoles are metabolized to IS and IDG in the liver [13]. IAA is metabolized directly in the intestine [14] or in tissue via tryptamine [11]. IS, IAA, and IDG are commonly excreted in urines and accumulate in case of CKD [5,15]. 

## 3. AhR Activation by Toxins Derived from Tryptophan Metabolism

AhR is a ligand-activated transcription factor involved in biological detoxication of ligands. It is a member of the family of basic helix-loop-helix-Per-ARNT-Sim (PAS) containing transcription factors. AhR ligands include a wide variety of ubiquitous environmental pollutants like halogenated aromatic hydrocarbons (HAH) such as 2,3,7,8-tetrachlorodibenzo-*p*-dioxin or dioxin (TCDD), polycyclic aromatic hydrocarbons (PAH) and co-planar polychlorinated biphenyls (PCBs) [16,17]. However, there are many naturally occurring ligands such as tryptophan derivatives [17]. Historically, the action of AhR was determined in the setting of chemical carcinogenesis. AhR binds to the promoters of AhR target genes such as cytochromes P450 (CYP) 1A1, CYP1B1, and AhR repressor, which is the inhibitor protein of AhR. AhR induces the expression of xenobiotic metabolizing enzymes, such as CYP genes, needed for the detoxication of AhR toxic ligands [18]. Prior to AhR activation, AhR is found in the cytoplasm, coupled with its chaperone proteins such as Hsp90 (heat shock protein90). When a ligand binds to AhR, the AhR/ligand/Hsp90 complex translocates into the nucleus and dimerizes with ARNT (Aryl hydrocarbon receptor nuclear translocator). The AhR/ARNT complex recognizes a DNA specific site, 5’-GCGTG-3’, the XRE (Xenobiotic Responsive Element) sequence in promoters of target genes, and induces their transcription [19,20]. A non-genomic inflammatory pathway of AhR activation that leads to other transcription factor activation such as NF-κB and AP-1 is also described [20]. The cytosolic AhR can activate a number of other cytosolic proteins, including proteins of the Mitogen-Activated Protein Kinase (MAPK) family Extracellular signal-regulated kinase (ERK), p38, and Jun-NH2-terminal kinase (JNK) [21,22,23,24,25]. AhR activation of MAPK depends on cell type. All three MAPKs phosphorylate downstream transcription factors and thereby alter gene expression to direct a specific cellular response. The AhR ligand TCDD induces ERK1/2 and p38 phosphorylation in rodent hepatoma cells [22] and in a mouse leukocyte cell line [23,24] and promotes JNK phosphorylation in a breast cancer cell line, MCF7 [25]. Furthermore, because the tyrosine kinase c-Src can be associated with cytosolic AhR complex, AhR activation by TCDD can also indirectly induce the MAPK cascade via c-Src [26,27]. C-Src phosphorylation results in EGFR phosphorylation, which triggers MAPK signal transduction and enhances the expression of target genes [27]. AhR also interacts with transcription factors such as AP-1 [24] and NF-κB, notably the RelA subunit. The interaction between AhR and NF-κB can lead to activation or mutual transrepression of their activities, depending on the duration, the cell type used, and the promoter being assayed [28,29]. These pathways were described in the setting of chemical carcinogenesis, their relevance in cardiovascular physiopathology has to be demonstrated.

Uremic toxins derived from tryptophan activate the AhR pathway. This was notably demonstrated for indolic toxins IS [6,30] and IAA [7,30], at uremic concentrations, in hepatic cells [6] and endothelial cells [30]. IS and IAA induce the nuclear translocation of AhR [30]. Using microarray analyses of endothelial cells, we found that IS and IAA induce an upregulation of eight genes regulated by AhR, including CYP1A1 and CYP1B1 [30]. The other uremic toxins derived from tryptophan metabolism described as AhR ligands are from the kynurenine pathway. In glioma cells, kynurenine induces AhR translocation into the nucleus and activates AhR target genes [31,32], but the concentrations of kynurenine used in those experiments are at least 10 times higher than those reported in uremic serum. For human NK cell lines, Shin *et al.* reported AhR activation by kynurenine [33] at uremic concentration [10,34]. Dinatale et al found that kinurenic acid can stimulate the AhR pathway [35] at concentrations found in CKD patients [10,34].

## 4. Involvement of AhR-Activating Uremic Toxins in Cardiovascular Diseases

The cardiovascular toxicity of AhR ligands was first described with environmental pollutants like TCDD and co-planar PCBs. A recent meta-analysis highlighted a consistent association between TCDD exposure and increased risk of cardiovascular mortality, especially from ischemic diseases [36]. Moreover, people exposed to co-planar PCBs present an increased risk of acute myocardial infarction and ischemic stroke [37]. An association between plasma concentration of PCB serum level and hypertension was reported in the general population [38]. Furthermore, PCB levels in serum are strongly associated with the number of atherosclerotic plaques in carotid arteries [39]. In rodents, chronic TCDD or PCB exposure increases cardiomyopathy [40,41] and systemic blood pressure [41], especially in female rats [42]. In an atherosclerosis model of apoE-/- mice, PCB or TCDD exposure leads to a higher incidence of atherosclerotic lesions [43,44,45].

The role of AhR-activating uremic toxins in cardiovascular disease, and notably the role of IS, has been described. Serum IS is associated with overall and cardiovascular mortality in CKD patients (stage 2 to hemodialyzed stage 5) [46], and with all-cause mortality in incident hemodialyzed patients [47]. IS is associated with coronary lesions in non-hemodialyzed diabetic CKD patients even after eGFR adjustment [48]. IS is also associated with left ventricular diastolic dysfunction in CKD non-hemodialyzed patients [49]. In a model of salt-sensitive hypertensive rat, increased IS serum levels stimulate cardiac fibrosis [50]. The role of IS is supported by a decrease in myocardial fibrosis by AST-120, an oral charcoal adsorbent limiting indole absorption in the intestine, resulting in decreased IS serum levels [51]. In subtotally nephrectomized apoE-/- mice, AST-120 improves atherosclerotic lesions and reduces IS deposition in aorta [52]. 

Uremic toxins from the kynurenine pathway may also play a role in cardiovascular diseases. Higher levels of 3-hydroxykynurenine are most frequent in patients with cardiovascular diseases [53]. Interestingly, for patients with recent ischemic stroke, Darlington et al found an association between serum kynurenic acid levels and patient mortality at 24h [54]. In CKD patients, the TRP/KYN ratio, which inversely correlates with kidney function [34], is related to carotid intima-media thickness, a presymptomatic predictor of atherosclerosis [55,56]. In these patients, higher IDO activity is associated with larger carotid plaques [57]. 

## 5. AhR-Activating Uremic Toxins Induce Endothelial Dysfunction

Endothelial dysfunction is reflected by impaired endothelium-dependent vasodilatation. In vivo, flow-mediated dilatation (FMD) is a good clinical marker of endothelial dysfunction and is associated with cardiovascular events [58,59]. AhR activation by environmental ligands inhibits endothelium-dependent vasodilatory response via an induction of CYP1A1 in mice [41,60]. In CKD patients, the FMD decreases with kidney function [61], and one can suppose that AhR-activating uremic toxins impair the endothelium-dependent vasodilatation. Indeed, the decrease in IS plasma level after the administration of oral AST-120 in CKD patients is associated with improved FMD [62]. Furthermore, administration of AST-120 in rats with subtotal nephrectomy improves acetylcholine-dependent vasodilatation of the aorta, in a dose-dependent manner [63]. 

Environmental pollutants TCCD and co-planar PCBs that induce AhR activation have deleterious effects on endothelial cells *in vitro*. The AhR agonist 3-methylcholanthrene inhibits endothelial cell adhesion, migration, and proliferation [64] through p38 MAPK phosphorylation; PCB126 decreases endothelial nitric oxide (NO) production [65]; 3-methylcholanthrene decreases endothelial stress fiber formation [66]; and PAHs decrease endothelial progenitor cell number and colonies [67]. All these mechanisms are also observed for uremic toxins derived from tryptophan. In vitro, IS inhibits endothelial proliferation and wound repair [68] and increases cell senescence [62]. IS also inhibits endothelial cell migration by a tardive inhibition of ERK1/2 phosphorylation [69]. IS decreases endothelial NO production [62], and the inhibition of endothelial cell migration is reversed by NO [69]. IS induces the endothelial cell adherens junction disassembly and stress fiber reorganization mediated by ERK1/2 phosphorylation that is induced by oxidative stress overproduction [70]. In CKD patients, IAA serum level is negatively correlated with the number of immature progenitor cells [71], and IAA induces progenitor cell apoptosis *in vitro* [71].

## 6. AhR-Activating Uremic Toxins Increase Oxidative Stress in Cardiovascular Cells

The role of AhR activation in oxidative stress is supported by studies demonstrating that AhR activation by TCDD and PCB induces reactive oxygen species (ROS) production [41,72,73,74] in the aorta, heart, and kidney [41,60] and in endothelial cells [73] via CYP1A1 expression [65,72,74]. 

In CKD patients, high IS [62], kynurenine [53], 3-hydroxykynurenine [53], and KYN/TRP ratio [75] are all associated with oxidative stress markers. Furthermore, in a model of salt-sensitive hypertensive rat, increased IS serum levels enhance cardiac oxidative stress [50]. In CKD patients, administration of AST-120, which reduces IS serum level, decreases plasma levels of oxidative stress markers [62]. AST-120 improves aortic [63] and cardiac [51] oxidative/nitrative stress in CKD rat models. In vitro, AhR-activating indolic uremic toxins such as IDG [76], IS [77], and IAA [personal data] induce endothelial ROS production. Induction of endothelial ROS production by IS occurs through activation of NADPH oxidase and inhibition of antioxidant defense mechanisms [76,77]. The link between a pro-oxidant effect of IS and AhR activation is supported by experiments demonstrating that the inductions of both Nox4 gene expression [78] and superoxide production by IS are inhibited by the AhR inhibitors ANF and CH-223191 [78]. 

In vascular smooth muscle cells (VSMC), IS stimulates NADPH oxidase-dependent ROS generation, promotes osteoblast differentiation [79], and induces VSMC senescence through oxidative stress pathway [80,81]. IS promotes the migration of VSMCs by a mechanism that involves ROS production [82,83] and phosphorylation of ERK1/2 [82,84] and p38 [82]. The involvement of AhR activation in the effects of IS on VSMC has not been studied. However, effects of IS mimic those of the AhR agonist Benzo [a]pyrene, which promotes VSCM migration [85] and ROS production [85,86] in an AhR-dependent manner.

## 7. AhR-Activating Uremic Toxins Induce Leukocyte Activation and Inflammation

The involvement of AhR activation in inflammation was demonstrated using environmental pollutants. In mice, PCB and TCDD exposure induces the expression of proinflammatory cytokines [45] and aortic expression of monocyte chemoattractant protein-1 (MCP-1) [87,88], but not in mice invalidated for AhR nuclear translocation [88]. In T cell immune response, TCDD induces lymphocyte activation via modifications in dentritic cell phenotype and functions [89]. Furthermore, AhR participates in T cell differentiation [90]. Exposure to AhR-activating environmental pollutants increases monocyte adhesion [91,92]. Macrophages treated with TCDD show an up-regulation of inflammatory genes IL-8 and MMP-12 and of VEGF in an AhR- and IL-8-dependent manner [45]. In vitro, AhR activation by PCB induces MCP-1 expression [73,87,91,93] through a ROS/NF-κB pathway [73]. PCB induces the expression of endothelial adhesion molecules E-selectin [91,94], VCAM-1 [92,94], and ICAM-1 [89,91,95] via MAPK signaling pathways [92,95]. Finally, AhR agonists increase endothelial COX-2 expression and COX-2 metabolites in urine [43,65].

A growing body of data supports the hypothesis that AhR activation by tryptophan-derived uremic toxins is involved in inflammation associated with CKD. Kynurenine and 3-hydroxykynurenine levels correlate with CRP in dialysis patients [53], and KYN/TRP ratio is positively associated with markers of inflammation in CKD patients [75]. Kynurenine and its metabolites induce T cell differentiation [96], and kynurenic acid activates IL6 in MCF-7 cells in an AhR-dependent manner [35]. IS stimulates inflammatory pathways, notably the p38/NF-KB pathway that leads to IL-6 and IL-1β expression [97,98]. IS enhances leukocyte adhesion in a subtotal nephrectomy mouse model after vascular lesion [99]. IS also enhances leukocyte adhesion to the endothelium *in vitro* by a ROS/p38 MAPK pathway [98] and a ROS/JNK/NF-κB pathway [99]. In monocytes, IS induces ROS production in an NADPH oxidase-dependent manner [98]. Furthermore, IS induces endothelial E-selectin [99], VCAM-1 [100], and ICAM-1 [100,101] expression. IS also induces the expression of MCP-1 [78,100,101,102], which is abolished by an AhR antagonist [78]. MCP-1 induction by IS involves a ROS/MAPK/NF-κB pathway [101,102], which evokes the involvement of the inflammatory non-genomic pathway of AhR. Finally, we have observed that IS and IAA induce COX-2 expression in endothelial cells [30] in an AhR-dependent manner [103]. 

## 8. Involvement of AhR-Activating Uremic Toxins in Thrombosis

AhR activation by tryptophan-derived uremic toxins could be involved in thrombosis. In hemodialyzed patients, the KYN/TRP ratio is positively associated with thrombosis markers [75], and IS level correlates with vascular access intervention [104]. In CKD patients, the levels of the indolic uremic toxins IS and IAA correlate with the levels and activity of tissue factor [30], the main initiator of the blood coagulation cascade. In endothelial cells and in peripheral blood mononuclear cells, IS and IAA increase tissue factor expression and tissue factor-dependent procoagulant activity [30]. Using siRNA and a pharmacological AhR inhibitor, we demonstrated that AhR activation is involved in tissue factor production induced by indolic uremic toxins [30]; we also showed that the canonical AhR agonist TCDD induces endothelial tissue factor expression [30]. The increase in tissue factor activity induced by IS and IAA is associated with enhanced clot formation by a pathway encompassing inhibition of tissue factor ubiquitination, which increases tissue factor half-life [105]. Interestingly, AHR was reported to interact with ubiquitination machinery [106].

## 9. Is AhR Activation Involved in CardioVascular Toxicity of CKD?

AhR can be activated by structurally diverse chemicals. The dramatic variety of effects produced by TCDD and other AhR ligands suggests significant mechanistic diversity in the action of these chemicals [20]. There are species- and tissue-specific toxic effects [20]. AhR-dependent toxic and biological effects are modulated by the structure and metabolism of AhR ligands leading to differences in AhR-dependent gene expression profiles [107]. TCDD results in a persistent activation of AhR due to its accumulation in adipose tissue [44,108] whereas other AhR ligands such as benzo [a]pyrene are rapidly metabolized and fail to induce sustained AhR activation. Little is known about the metabolism and the tissue distribution of tryptophan-derived uremic toxins in CKD. However the accumulation of these uremic toxins in patients suggests the possibility of a persistent activation of AhR. The hypothesis that AhR activation is involved in the cardiovascular toxicity of tryptophan-derived uremic toxins has to be confirmed in animal and human studies.

## 10. Conclusions

Tryptophan-derived uremic toxins share, with environmental pollutants like dioxins, the ability to activate the AhR pathway. These toxins, like environmental pollutants, induce endothelial dysfunction and leukocyte activation, favor inflammation and thrombosis, and increase vascular oxidative stress (Table 1). The ability of tryptophan derived of uremic toxins to activate AhR may explain how these toxins contribute to cardiovascular diseases in CKD patients. These recent insights into the mechanisms of toxicity of tryptophan-derived uremic toxins may lead to new therapeutic strategies targeting AhR activation.

**Table 1:** Similarities between the effects of aryl hydrocarbon receptor (AhR)-activating pollutants and the effects of tryptophan-derived uremic toxins.

Effects of AhR-activating-pollutants	Effects of tryptophan-derived uremic toxins
Association with cardiovascular events	
Association with cardiovascular mortality [36] Association with higher hypertension [37] Association with hospitalization for ischemic stroke [37]	Association with cardiovascular mortality [46] Association with overall mortality [46,47] Association with the mortality of ischemic stroke [54]
Associated with number of atherosclerotic carotid plaques [39]	Association with greater size of atherosclerotic carotid plaques [57] Association with coronary lesions [48]
Decrease in endothelial-dependent vasodilation [41,60]	Decrease in endothelial-dependent vasodilation [62,63]
Induction of cardiomyopathy in rodents [40,41] Higher incidence of atherosclerotic lesions in mice [43,44,45]	Induction of cardiac fibrosis in rodents [50,51] Induction of atherosclerotic lesions in mice [52]
Endothelial dysfunction	
Inhibition of endothelial cell proliferation [64] Inhibition of endothelial NO production [65] Inhibition of endothelial cell migration [64] Decrease in endothelial progenitor cells [67] Induction of stress fiber formation [66]	Inhibition of endothelial cell proliferation [62,68] Inhibition of endothelial NO production [62] Inhibition of endothelial cell migration [69] Decrease in progenitor cells [71] Induction of stress fiber reorganization [70]
Oxidative stress	
Association with oxidative stress markers [41,60,72,73] Induction of endothelial ROS [65,72,73,74] Induction of VSMC migration by ROS [85,86]	Association with oxidative stress markers [53,62,75] Induction of endothelial ROS [76,77] Induction of VSMC migration by ROS [82,83]
Thrombosis	
Induction of TF production and activity [30]	Induction of TF production and activity [30]
Inflammation	
Association with inflammatory markers [45,88]	Association with inflammatory markers [53,75]
Increase in monocyte expression of inflammatory cytokines [45,89]	Increase in monocyte expression of inflammatory cytokines [97,98]
Increase in monocyte adhesion [92,94]	Increase in monocyte adhesion [98,99]
Induction of VCAM-1 [92,100], MCP-1 [73,87,91,93], E-selectin [91,94] ICAM-1 [89,91,95] and COX-2 [65]	Induction of VCAM-1 [100], MCP-1 [78,100,101,102], E-selectin [99], ICAM-1 [100,101] and COX-2 [30]
Induction of urinary COX-2 metabolites in mice [43]	
Induction of T cell differentiation [90]	Induction of T cell differentiation [96]
